# Role of Acamprosate and Baclofen as Anti-craving Agents in Alcohol Use Disorder: A 12-Week Prospective Study

**DOI:** 10.7759/cureus.58174

**Published:** 2024-04-13

**Authors:** Akhilesh K Sharma, Praveen Rikhari, Alok K Shukla, Pragya Rikhari

**Affiliations:** 1 Psychiatry, Mental Hospital Bareilly, Bareilly, IND; 2 Department of Psychiatry, Himalayan Institute of Medical Sciences, Swami Rama Himalayan University, Dehradun, IND; 3 Department of Statistics, Himalayan Institute of Medical Sciences, Swami Rama Himalayan University, Dehradun, IND

**Keywords:** craving, relapse, baclofen, acamprosate, pacs, alcohol use disorder

## Abstract

Background

Alcohol use disorder (AUD) is one of the most common substance use disorders globally. It is a chronic mental illness characterized by frequent relapses. Hence, preventing relapse is one of the most important aspects of the management of patients with AUD.

Aims

This study aimed to compare the role of acamprosate and baclofen as anti-craving agents in patients diagnosed with AUD.

Settings and design

This was a 12-week interventional follow-up study conducted in the Department of Psychiatry of S N Medical College, a tertiary care teaching hospital in Agra, Uttar Pradesh, India.

Methods and materials

Patients with AUD were enrolled in the study. Following medical management of alcohol withdrawal symptoms, patients were alternately assigned to receive either acamprosate or baclofen and were then followed up for 12 weeks. Measures to compare the effectiveness of the two medications were craving as measured using the Penn Alcohol Craving Scale (PACS), days to first alcohol consumption, days to relapse, number of drinks consumed at one occasion, number of patients who completed the study, and number of patients who remained abstinent throughout the duration of the study. Descriptive statistics were used to present the data while unpaired t-test and Fisher’s exact test were used to compare the two groups.

Results

A total of 63 patients were enrolled in the study. Following medical management of alcohol withdrawal symptoms for one week, 50 (79.37%) patients were retained in the study. Hence, these 50 patients were assigned to treatment with either acamprosate or baclofen alternately in a 1:1 ratio. Only 32 (64%) of the patients who were started on these medications completed the study and were available for analysis at the end of 12 weeks. Acamprosate-treated patients were found to have less severe cravings (p < 0.01) for alcohol at the end of the study and also had consumed less number of drinks on a single occasion (p < 0.05). For other variables being considered in the study, namely, days to first alcohol consumption, days to relapse to previous drinking pattern, number of patients who dropped from the study versus those who completed the study, and those who were abstinent versus those who relapsed, no statistically significant difference was noted.

Conclusion

Acamprosate-treated patients had significantly lesser cravings for alcohol and consumed a lesser number of drinks on one occasion compared to baclofen-treated patients in this 12-week study.

## Introduction

Alcohol, chemically ethanol, is the psychoactive component of beverages like beer, wine, and distilled spirits. It is manufactured through the fermentation of sugars present in fruits, grains, and other sources.

Alcohol use is widely prevalent throughout the world. According to the Global Burden of Disease survey, alcohol use disorder (AUD) is one of the most common substance use disorders worldwide with about 2.4 billion people or 33% of the world's population being current drinkers. It is also a leading cause of mortality and morbidity globally [1]. In India, about 160 million people or 14.60% of the total population use alcohol. Out of them, 57 million are problem users and 29 million are dependent users [2].

The Diagnostic and Statistical Manual of Mental Disorders-5 (DSM-5) defines AUD as a syndrome resulting from chronic problematic alcohol use that continues despite adverse physical, psychological, interpersonal, and social consequences. It also leads to a cluster of behavioral and physical symptoms, which include withdrawal, tolerance, and craving [3].

Alcohol-dependent patients experience intense cravings for alcohol when abstinent and remain at a high risk of relapse especially during the first few months after detoxification [4]. Different pharmacological agents have been studied to reduce craving and prevent relapse in these patients. Acamprosate, an N-methyl-D-aspartate (NMDA)-receptor antagonist is FDA-approved for its anti-craving effects in AUD [5]. Another drug under exploration for its anti-craving effects is baclofen, which is hypothesized to exert its effects by reducing dopamine release via GABA-B receptor activation [5,6].

In this study, we analyzed the anti-craving effects of acamprosate and baclofen over 12 weeks in patients with AUD.

## Materials and methods

This was a randomized clinical study with purposive sampling conducted as a thesis project at the Department of Psychiatry of S N Medical College, a tertiary care teaching hospital in Agra, Uttar Pradesh, India. All patients attending psychiatry services from January 2019 to January 2020 were evaluated for eligibility to participate in the study. The inclusion criteria were being diagnosed as a case of AUD according to DSM-5, age between 18 and 60 years, and individuals who gave written informed consent. The exclusion criteria were an abnormal kidney function test, acute biliary disease, hemolytic anemia, positive viral markers (for hepatitis B, hepatitis C, or human immunodeficiency virus), any other chronic medical illness, comorbid current psychiatric disorder, use of antiepileptic and anti-tubercular medications, and being pregnant or breastfeeding.

The Alcohol Use Disorders Identification Test (AUDIT) was used for screening patients at baseline. It is a 10-item screening tool developed by the World Health Organization to assess alcohol consumption, drinking behaviors, and alcohol-related problems. The range of possible scores is from 0 to 40, where 0 indicates an abstainer who has never had any problems with alcohol. A score of 1 to 7 suggests low-risk consumption, scores from 8 to 14 suggest hazardous or harmful alcohol consumption, and a score of 15 or more indicates the likelihood of alcohol dependence (moderate-severe AUD). Scores of 8 or above were used to identify the patients eligible for the study [7]. The Severity of Alcohol Dependence Questionnaire (SAD-Q) scale was applied to assess and quantify the severity of alcohol dependence. This is a 20-item clinical tool designed to measure the presence and level of alcohol dependence based on alcohol consumption patterns and experiences after drinking alcohol. The severity of alcohol dependence is classified as mild dependence (SAD-Q score 8-15), moderate dependence (SAD-Q score 16-30), and severe dependence (SAD-Q score 31-60) [8]. The Penn Alcohol Craving Scale (PACS) was used to quantify craving for alcohol during follow-up. It is a self-report measure with five items that includes questions related to frequency, intensity, and duration of craving, the ability to resist drinking, and an overall rating of craving for alcohol in the previous week. Each item is scored from 0 to 6, and the maximum total score can be 30 [9].

Medical management of alcohol withdrawal in these patients was done using lorazepam (2-12 mg), which was tapered over seven days along with co-administration of 300-500 mg of thiamine per day in divided doses. In addition, vitamin C and vitamin B complex including folic acid were given. 

After the management of acute symptoms of alcohol withdrawal over seven days, patients were divided into two groups with the first patient receiving acamprosate, the second patient receiving baclofen, the third receiving acamprosate, and so on in an alternate manner. Baclofen was dosed at 30 mg/day in a single dose as an extended-release formulation, similar to studies by Rombouts et al. and Morley et al. [10,11]. The dosage of acamprosate was 1,998 mg/day (if weight ≥60 kg) and 1,332 mg/day (if weight <60 kg) in three divided doses based on previous literature [12]. The anti-craving effects of these agents were compared using the mean PACS score between the two treatment groups at 12 weeks. Other variables compared between the two treatment groups were days to first alcohol intake, days to relapse to previous alcohol consumption patterns, and the average number of drinks of alcohol (one drink of alcohol is equivalent to eight grams of pure alcohol) consumed on one occasion after being enrolled in the study. 

The dataset was verified, validated, and analyzed using IBM SPSS Statistics for Windows, version 27.0 (released 2020, IBM Corp., Armonk, NY). Descriptive statistics were applied, categorical data were presented as frequency and percentage, and quantitative data were presented as mean with standard deviation. An unpaired t-test was used to evaluate the difference between the two groups in terms of mean craving scores at 12 weeks as measured using PACS, mean days to first alcohol consumption, mean days to relapse, and mean number of drinks consumed on one occasion. Fisher's exact test was used to compare the number of patients who dropped out of the study versus those who completed the study and the number of subjects who were abstinent throughout the study versus those who relapsed between the two groups. A p-value of less than 0.05 was considered to be statistically significant.

This study was a dissertation project and was approved by the Institutional Ethics Committee of Sarojini Naidu Medical College, Agra, Uttar Pradesh, vide letter no. IEC/2021/50.

## Results

All the study participants were males. About half of the participants were between the age of 31-40 years. The majority were Hindus, married, living in an extended family, residing in an urban area, belonging to the upper lower class or below, having education equal to or less than the secondary level, and having a family history of alcohol use (Table 1).

**Table 1:** Sociodemographic characteristics of the study sample

Sociodemographic characteristics		Baseline (n = 63)
Gender	Male	63 (100%)
Age (in years)	≤30	12 (19.05%)
	30-40	32 (50.79%)
	≥40	19 (30.16%)
Religion	Hindu	53 (84.13%)
	Muslim	10 (15.87%)
Locality	Urban	50 (79.37%)
	Rural	13 (20.63%)
Marital status	Married	58 (92.06%)
	Unmarried	5 (7.94%)
Education	Up to secondary	44 (69.84%)
	Intermediate or above	19 (30.16%)
Socioeconomic status	Lower middle or above	27 (42.86%)
	Upper lower or below	36 (57.14%)
Family history of alcohol use	Yes	42 (66.67%)
	No	21 (33.33%)
Family type	Extended	37 (58.73%)
	Nuclear	27 (41.27%)

Using the SAD-Q scale, 20 (32%) were found to have mild alcohol dependence, 22 (35%) patients were found to have moderate alcohol dependence, and 21 (33%) patients were found to have severe alcohol dependence (Table 2).

**Table 2:** Severity of alcohol dependence in the study sample

Severity of alcohol dependence	Number of participants (n = 63)
Mild	20 (31.75%)
Moderate	22 (34.92%)
Severe	21 (33.33%)

Out of the total 63 (100%) patients enrolled in the study, 50 (79.37%) were adherent on the seventh day, and 32 (50.79%) were available for analysis at the end of 12 weeks. Thus, only about half of the patients (50.79%) were available for analysis at week 12.

A total of 50 (79.37%) patients were available for the initiation of anti-craving medications after a detoxification period of 1 week, of whom one-half received acamprosate and the other half received baclofen.

Only 18 (72%) patients from the acamprosate group and 14 (56%) patients from the baclofen group followed up for the total duration of the study, which was 12 weeks. When compared using Fisher’s exact test, no statistically significant difference was found between the two groups in terms of the number of patients who dropped out of the study versus those who completed the study (p = 0.33) (Table 3).

**Table 3:** Number of subjects who completed the study versus those who dropped out in the two groups

Parameters	Acamprosate (n = 25)	Baclofen (n=25)	p-value
Subjects who dropped out of the study	7 (28.00%)	11 (44.00%)	0.33
Subjects who completed the study	18 (72.00%)	14 (56.00%)	

At the end of the study, seven (38.89%) subjects were abstinent from alcohol in the acamprosate group and three (21.43%) subjects were abstinent from alcohol in the baclofen group, whereas the rest of the subjects relapsed in both groups. When compared using Fisher’s exact test, no statistically significant difference was present between the two groups for the number of patients who were abstinent throughout the study versus those who relapsed during the study (p = 0.44) (Table 4).

**Table 4:** Number of subjects who were abstinent from alcohol versus those who relapsed in the two groups

Parameters	Acamprosate (n = 18)	Baclofen (n = 14)	p-value
Subjects who were abstinent throughout the study	7 (38.89%)	3 (21.43%)	0.44
Subjects who relapsed during the study period	11 (61.11%)	11 (78.57%)	

The mean PACS score between acamprosate and baclofen-treated patients was calculated for all the patients who completed the study and was compared using the unpaired t-test. In the patients who restarted taking alcohol during the study period, we applied the unpaired t-test to compare the mean days to first alcohol consumption, the mean number of days to relapse, and the mean number of drinks consumed on one occasion.

The mean PACS score in patients receiving acamprosate was 7.10, and the mean PACS score in patients receiving baclofen was 11.07. The mean days to first alcohol consumption and mean days to first relapse were 29.91 and 37.64 in the acamprosate group versus 11.01 and 25.00 in the baclofen group. The mean number of drinks consumed on one occasion was 5.45 in the acamprosate group and 8.82 in the baclofen group. There was a statistically significant difference in the mean PACS score (p = 0.006) and the mean number of drinks consumed on one occasion (p = 0.01) between the two treatment groups. The patients on baclofen consumed more drinks on one occasion and had greater cravings for alcohol after 12 weeks of treatment. On the other hand, no statistically significant differences for mean days to first alcohol consumption (p = 0.07) and mean days to the first relapse (p = 0.14) were noted between the two treatment groups (Table 5 and Table 6).

Post-hoc analysis on G*Power software (Universitat Kiel, Germany) was done using the mean score of craving as measured by the PACS as an outcome measure, and a power of 0.80 was detected, keeping α as 0.05 and using an effect size as 1.06.

**Table 5:** Comparison of alcohol craving as measured by PACS at 12 weeks between acamprosate- and baclofen-treated subjects * p-value significant at <0.05. PACS: Penn Alcohol Craving Scale

Parameters	Acamprosate (n = 18)	Baclofen (n = 14)	p-value
Craving severity at week 12 (PACS score) (mean ± SD)	7.10 ± 3.75	11.07 ± 3.73	0.006*

**Table 6:** Comparison of outcomes directly related to alcohol consumption between acamprosate- and baclofen-treated subjects * p-value significant at <0.05

Parameters	Acamprosate (n = 11)	Baclofen (n = 11)	p-value
Days to first alcohol consumption (Mean ± SD)	29.91 ± 18.56	11.01 ± 1.88	0.073
Days to the first relapse (Mean ± SD)	37.64 ± 21.50	25.00 ± 14.22	0.144
Number of drinks consumed on one occasion (Mean ± SD)	5.45 ± 2.25	8.82 ± 3.28	0.011*

## Discussion

In our study, all subjects were males, similar to studies conducted by Sarkar et al., Mohandas et al., and Sujiv et al. [13,14,15]. This may occur due to various factors, such as social structure, financial dependence on others, and societal attitudes toward female drinkers, which may make seeking help less likely or difficult for females.

A higher representation of patients from lower socioeconomic strata is probably due to the study being conducted in a state-run institution providing treatment to patients at a minimal cost. Similarly, most of the patients had received education up to the secondary level, in line with the predominance of participants from the lower strata of society. 

About one-third of those who started on treatment were lost to follow-up over the course of the study. These high rates of loss to follow-up may be the reflection of the relapsing nature of AUD itself. This was a non-sponsored, non-funded study with limited resources, which might also have contributed to the high attrition rate. Some other contributory factors might be perceived good health and self-perception of being able to abstain from alcohol without medical help. In addition, no outreach programs or telephone-based services were used to ensure follow-up. 

The treatment outcomes between acamprosate and baclofen were compared. The number of patients who relapsed, mean days to first alcohol consumption, and mean days to first relapse showed no significant difference between the two drugs but the number of drinks consumed on one occasion was significantly lesser in the acamprosate group compared to the baclofen group. The mean PACS score was also significantly lesser in the acamprosate group. Although not statistically significant numerically, a greater percentage of patients on acamprosate completed the study with fewer dropouts compared to baclofen. One possible conclusion might be that acamprosate is better tolerated than baclofen in patients of AUD, although a number of other factors may be at play. The lack of any significant difference between the patients treated with these two medications for the percentage of patients who relapsed during the study period reflects that both may have similar efficacy in preventing relapse to previous drinking patterns. The high percentage of patients who relapsed also reflects the relapsing nature of AUD.

We found that patients who were being treated with acamprosate had less severe cravings at 12 weeks compared to patients who were being treated with baclofen. This implies that in reducing the craving for alcohol, acamprosate is better and should be preferred in patients who report greater craving for alcohol. The relatively more number of days for first alcohol intake and relapse to previous patterns of drinking alcohol in patients treated with acamprosate can be explained by the finding of lesser craving in them, as consequent to lesser craving, they might have been able to resist the urge to take alcohol for a longer duration. Therefore, craving can be considered to be an important mediator of restarting and increasing alcohol use in patients with AUD. Similarly, the significant difference between the two treatment groups in terms of the number of drinks consumed on one occasion can be explained by reduced craving and the ability to resist urges to take greater amounts of alcohol in patients treated with acamprosate. 

Some other similar studies have been conducted for preventing relapse in AUD. One is a study conducted by Kumar et al. who compared patients receiving baclofen or acamprosate for AUD. They found that patients treated with baclofen had less severe cravings, less percentage of dropouts from the study, but a greater rate of relapse of alcohol use compared to the acamprosate group [16]. 

The rates of abstinence and study completion for the acamprosate group in our study were similar to the study by Rubio et al. who compared the anti-craving effects of naltrexone and acamprosate [17]. A study by Desousa et al. comparing outcomes between acamprosate and disulfiram found acamprosate to be superior in terms of the number of patients completing the study, number of patients remaining abstinent, number of patients who relapse, mean days to first alcohol consumption, mean days to first relapse, and number of drinks consumed at one time [18].

Morley et al. compared baclofen as an anti-craving agent with a placebo in a 12-week study and did not find any significant difference between the baclofen-treated group and placebo-treated group for the number of days to relapse, number of days to first alcohol consumption, and number of drinks consumed per drinking day [19]. On the other hand, Rombouts et al. found that relative to a placebo, baclofen increased time to first drink and time to first relapse in patients with higher alcohol consumption at baseline [10]. Addolorato et al. in their placebo-controlled study noted that baclofen significantly suppressed the mean number of daily drinks compared to the placebo [20].

Since patients with AUD are commonly seen in clinical practice, these findings can be helpful in deciding treatment plans for such patients. Therefore, acamprosate appears to be relatively better than other agents including baclofen in terms of anti-craving effects for some measures although variable findings do exist. For many other variables related to relapse of alcohol use, significant differences might not exist between the drugs. Oftentimes, a combination of drugs might also be warranted. 

The strengths of this study are its longitudinal design and measurement of craving using a standard scale, i.e., PACS, along with other variables that may determine the long-term outcome and risk of relapse in patients with AUD.

The limitations of the study include the small sample size with a high dropout rate as we did not use any method to ensure follow-up, short duration of the study, absence of a control group and placebo, potential clustering of severely addicted ones in one group, and lack of any method to ensure compliance with the prescribed drugs. Furthermore, the duration of alcohol intake, alcohol-related medical illnesses, prior unsuccessful attempts at abstinence, the severity of withdrawal, and social factors were also not taken into account.

Future studies can be planned from multiple sites with larger sample sizes, longer follow-ups, and using placebo and control groups.

## Conclusions

Preventing relapse is one of the most important components of the long-term management of AUD. Through this study, the authors intended to compare two drugs that have been used as long-term maintenance treatment in order to prevent relapse of alcohol use. This study was a 12-week follow-up study in patients with AUD who after medical management of acute withdrawal were prescribed either acamprosate or baclofen as maintenance therapy to prevent relapse of alcohol use. The study compared patients maintained on these two drugs on different parameters like cravings for alcohol at the end of 12 weeks, number of patients retained in the study, number of patients abstinent from alcohol, number of days to first intake of alcohol, and number of days to relapse during the study period. 

We found that patients with AUD who were prescribed acamprosate reported less severe cravings for alcohol and reported consuming less number of drinks on one occasion. For the rest of the variables under study, no significant association was noted. Hence, it was concluded that in patients with AUD, acamprosate can be a better option than baclofen in reducing cravings for alcohol and the amount of alcohol taken at one time.
